# Assessment of The Lymphatic System of the Genitalia Using Magnetic Resonance Lymphography Before and After Treatment of Male Genital Lymphedema

**DOI:** 10.1097/MD.0000000000003755

**Published:** 2016-05-27

**Authors:** Qing Lu, Zhaohua Jiang, Zizhou Zhao, Lianming Wu, Guangyu Wu, Shiteng Suo, Jianrong Xu

**Affiliations:** From the Department of Radiology (QL, ZZ, LW, GW, SS, JX), Renji Hospital, School of Medicine, Shanghai Jiao Tong University; and Department of Plastic & Reconstructive Surgery (ZJ), Shanghai 9th People's Hospital, School of Medicine, Shanghai Jiao Tong University, Shanghai, China.

## Abstract

Treatment for chronic male genital lymphedema (GL) is limited. No standard treatment or validated instrument to assess GL is available. The aim of this study was to explore whether magnetic resonance lymphography (MRL) could be used to assess GL, select proper treatment for patients, and monitor postoperative progress.

This is a retrospective analysis of a prospectively acquired cohort of men with GL presenting for MRL over a 7-year period. Thirty-six of 47 eligible men were included. All men were offered preoperative and postoperative MRL and assigned a morphology and function classification. Men with mild, moderate, and severe nodal dysfunction underwent complex decongestive physiotherapy (CDP), lymphoveneous microsurgery, and surgical excision, respectively. The volume reductions in the genitalia of patients with mild and moderate nodal dysfunction were recorded and compared using Student *t* test.

The abnormal superficial and deep lymphatic vessels in the lymphedematous genitalia were detected by MRL, and inguinal lymph node dysfunction was classified by MRL. Seven patients with mild dysfunction who underwent CDP showed a more significant mean volume reduction in the genitalia than did 9 patients with moderate dysfunction. Three patients with hyperplasia and moderate dysfunction who underwent microsurgical operations and 17 patients with hypoplasia and moderate or severe nodal dysfunction who underwent surgical excision had excellent cosmetic results with no lymphedema at the 3- to 5-year follow-up.

MRL can be used to assess morphological and functional lymphatic abnormalities in GL, preoperatively select appropriate treatment, and postoperatively evaluate treatment outcomes.

## INTRODUCTION

Genital lymphedema (GL) in men is a chronic disease that severely affects the patient and imposes a significant economic burden on the health system.^1^ It has been estimated that 20% of the male population in tropical countries suffers from scrotal lymphedema.^2^ In developed countries, however, scrotal lymphedema is usually secondary to surgery and radiation in the pelvic region.^3,4^ The condition can be disabling psychologically and physically. GL causes disfigurement, pain, and difficulties with urination and sexual function. Traditionally, treatment of this disease has been dependent on its etiology and clinical stage. For some patients with early-stage disease, conservative therapy is sufficient. For others in whom the disorder is disabling and persistent, a major reconstructive procedure is necessary. However, no standards exist for the treatment of GL because of the high recurrence rate.^2,5^ Patients who suffer from this disease are usually frustrated by repeated visits to multiple health care providers who are rarely able to provide proper care. Therefore, understanding the conditions underlying GL and performing the appropriate procedure for effective treatment are necessary.

Currently, there is no validated instrument available for treatment planning and the evaluation of treatment outcomes in GL. Since 2007, magnetic resonance lymphography (MRL) using the paramagnetic contrast agent gadobenate dimeglumine has been routinely used in our clinic to assess lymphatic system architecture and lymphatic drainage in extremities affected by lymphedema.^6–8^ MRL may have the potential to assess the groin lymph nodes and scrotal lymphatic vessels simultaneously, thereby enabling doctors to tailor a suitable procedure to the individual patient, thereby reducing morbidity.^9^ In this study, we prospectively evaluated our experience with MRL in patients with GL to assess its efficacy and to better understand the lymphedema evaluation process and the optimal patient selection for lymphedema treatment. We also present the follow-up MRL results in these patients after lymphedema treatment. To our knowledge, this is the first time MRL has been used to develop treatment strategies and to assess the long-term treatment outcomes in patients with GL.

## METHODS

###  Ethics Statement

This prospective study was approved by the institutional review board. Written informed consent was obtained from each participant before enrollment in the study.

###  Participants

**FIGURE 1:**
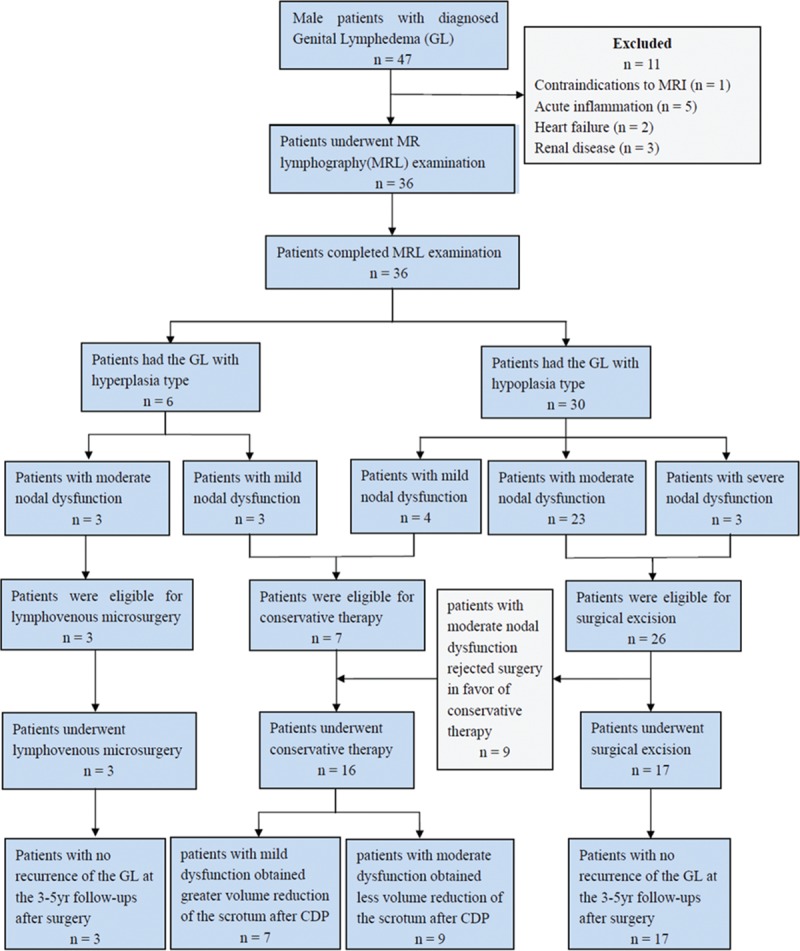
A chart to report flow of participants through the study.

From March 2008 to January 2015, 47 male patients with diagnosed GL were involved in the study. Eleven patients were excluded from this study because of contraindications for an MRI, genital swelling owing to acute inflammation, heart failure, and renal disease (Figure 1). Thus, there were 36 subjects enrolled, and the mean age was 52.3 ± 11.9 years (range: 27–74 years). The lymphedema characteristics are summarized in Table 1. The clinical diagnosis and staging were performed by a lymphologist (ZJ) with over 20 years of experience. The clinical staging was based on Casley-Smith's 3-part lymphedema staging system.^10,11^ All 36 patients underwent MRL examination before treatment.

**TABLE 1:**
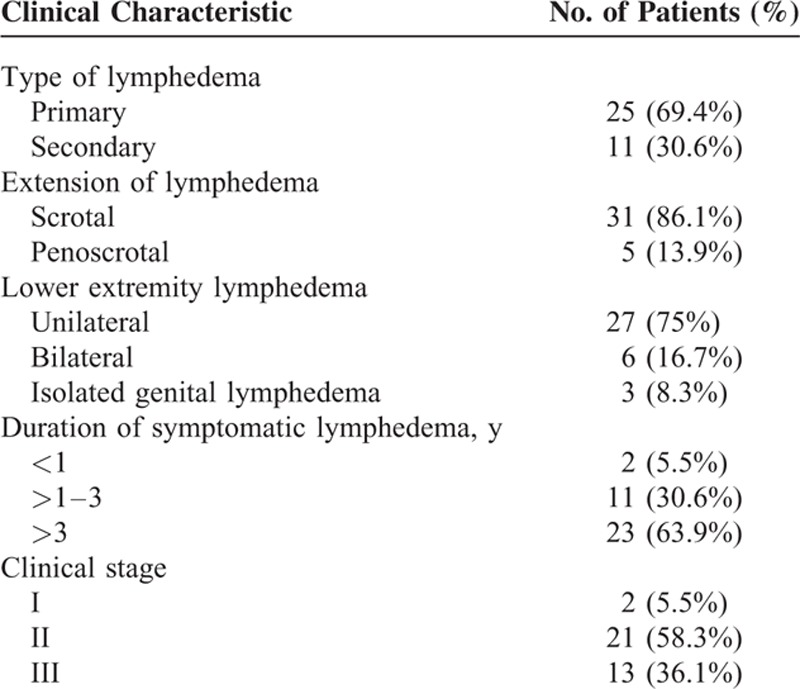
Clinical Characteristics of Genital Lymphedema in 36 Patients

###  MRL

All MR examinations were performed using a 3.0-T MR unit (Philips Medical System, Best, The Netherlands). A 6-channel phased-array sensitivity encoding cardiac reception coil was used. The scanning was performed in the inguinal region. For dynamic contrast-enhanced MRL, 3D fast spoiled gradient-recalled echo T1-weighted images with a fat saturation technique (TR/TE: 3.5/1.7, flip angle: 25, FOV: 375 cm × 320 cm, matrix: 750 × 640, voxel size: 1.2 mm × 0.5 mm, acquisition time: 3 minute) were acquired before and 3, 6, 9, 12, 15, 18, and 21 minutes after contrast injection. The contrast agent used for genital MRL is the commercially available and widely used paramagnetic contrast medium gadobentate dimeglumine (gadolinium benzyloxypropionic tetra-acetate, Gd-BOPTA, Multihance, Bracco, Torino, Italian). A mixture of 10% lidocaine and gadobentate dimeglumine (ratio, 1:10) was injected intracutaneously into the base of the scrotum, with 1 injection in each side. The volume injected into each point was 0.5 mL. After injection, a soft massage around the injection site was performed for 30 seconds.

###  Image Processing and Interpretation

The MR images were processed and evaluated at a dedicated MR workstation (Philips MR workspace 2.6.3.1, Best, The Netherlands) by 2 experienced radiologists who were blinded to the clinical characteristics of GL. The volumes of the scrotum and penis before and after treatment were calculated using MRL data via 3D reconstruction. To visualize the lymphatic vessels, the 3D MRL images were reconstructed from the post-contrast coronal images at each time point using a Maximum Intensity Projection technique. The appearance of the lymphatic vessels in comparison with that of the enhanced veins was described in our previous study.^6^ We defined the types of GL according to the number of the identified lymphatic vessels. If ≤4 superficial lymphatic vessels were observed, the disease was defined as hypoplasia. If ≥5 superficial lymphatic vessels were observed, the disease was defined as hyperplasia. To assess the lymphatic drainage function of the bilateral inguinal lymph nodes, the operator-defined regions of interest (ROIs) were drawn on the coronal pre- and post-contrast images. Time intensity curves (TICs) were constructed from signal intensity (SI) values obtained from the ROIs. Based on inguinal nodal enhancement TIC, we classified the function of the inguinal lymph nodes using 4 grades: normal function and mild, moderate, and severe nodal dysfunction. If the bilateral inguinal lymph nodes were obviously enhanced and had a peak enhancement of <9 minutes, we defined the nodal function as normal. Mild dysfunction was represented by bilateral inguinal lymph nodes that were simultaneously enhanced and 1 or/and 2 of the bilateral inguinal nodes that reached peak enhancement in over 12 minutes. Moderate dysfunction was represented by only 1 bilateral inguinal lymph node that showed peak enhancement and a time to peak enhancement >12 minutes. Severe dysfunction was represented by a lack of peak enhancement in the bilateral inguinal lymph nodes on MRL.

###  Treatment Strategy

The determination of the treatment modality was based on the lymphatic type of GL and the nodal function classification. Patients with normal nodal function or mild nodal dysfunction received conservative therapy. Patients with hypoplasia and moderate or severe nodal dysfunction underwent surgical excision and/or testis and penis reconstruction. Patients with hyperplasia and moderate or severe nodal dysfunction were subjected to lymphovenous microsurgery using deep lymphatic vessels. The different treatment measures chosen based on MRL findings were described in Figure 1. The patients who accepted surgical treatment were followed by MRL yearly for a 3- to 5-year period. The MRL was performed using the same protocol as before surgery.

###  Conservative Therapy

Conservative measures refer to the complex decongestive physiotherapy (CDP) first described by Foldi et al.^12,13^ The patients underwent intensive CDP at our specialized lymphological clinic for 4 weeks. This therapy involves manual lymph drainage twice daily, scrotal elevation with athletic support garments, physical exercises, and skin care. Noncontrast 3D MRL sequence was performed to evaluate the size change in the scrotum for patients who completed the CDP therapy.

##  SURGICAL APPROACH

###  Lymphovenous Microsurgery

**FIGURE 2:**
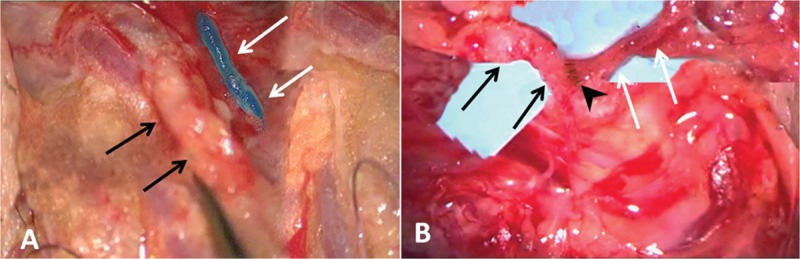
Lymphovenous microsurgery. (A) Under ×40 magnification, the targeted venule (black arrows) and deep lymphatic collector (white arrows) were identified using isosulfan blue dye and were prepared for anastomosis. (B) Lymphovenous terminolateral anastomoses were repaired using interrupted 9–0 polypropylene stitches (black arrow head).

Briefly, the microsurgical technique included the use of a subcutaneous injection of blue dye to visualize the lymphatic network and a small left curial incision identifies the spermatic funiculus and its elements. Two main deep lymphatic vessels were traced by vital blue along the spermatic cords. Using microscopic magnification (×40), lymphatic collectors of the spermatic funiculus (diameter approximately 0.5 mm) were prepared and anastomosed with venous branches of the pampiniformis plexus (Figure 2A). Lymphovenous side-to-side or end-to-side anastomoses were repaired using interrupted 9–0 or 10–0 polypropylene stitches (Figure 2B).The efficiency of the anastomosis was assessed by the absence of detectable blood back flow into the lymphatic collector and whether the size of the lymphatic collector remained constant for 30 minutes after the microsurgery.

###  Surgical Excision and Scrotal Reconstruction

**FIGURE 3:**
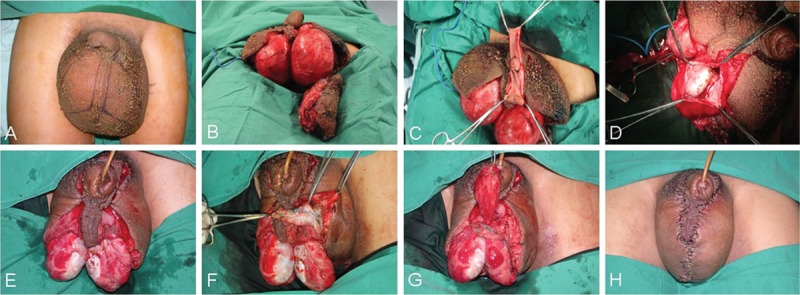
Clinical photographs for surgical excision and scrotal reconstruction. (A) The incision design retaining the scrotal septum. (B) The affected scrotal skin and subcutaneous edematous tissues were excised maximally. (C) Circumcision combined with removal of the subcutaneous edema tissue. (D) The testes and spermatic cords were isolated and the tunicae vaginalis were inverted. (E) The inverted tunicae vaginalis was fixed with inferior scrotal tissue and separated by the scrotal septum. (F and G) The subcutaneous lymphatic tissue flaps were traced using vital blue, and the remaining adjacent skin was bridged to the contralateral tissue. (H) Immediate postoperative period showing normal scrotal appearance.

The incision was designed to retain the scrotal septum and have direct contact with any normal-appearing skin. Based on the MRL results, the scrotal skin on the side with less nodal dysfunction was compared with that on the other side to reconstruct the scrotum following excision of the affected skin. Circumcision combined with removal of subcutaneous edematous tissue was used to treat penile lymphedema. The testes and spermatic cords were isolated, and the tunicae vaginalis were inverted. The subcutaneous lymphatic tissue flap traced by vital blue was preserved as much as possible and bridged by suture to the contralateral tissue. The remaining adjacent skin was used to cover the spermatic cords and testes, and a midline suture was tethered to the retained scrotal septum to reconstruct the scrotal raphe (Figure 3A–H). Postoperative rehabilitation consisted of affected lower limb lymphatic drainage and the long-term use of a scrotal suspender.

### Data Analysis

The Student *t* test was used to compare the differences between mean values. A *P* value of <0.05 indicated a significant difference.

## RESULTS

###  MRL

The examination time for 1 patient was approximately 35 minutes. No systemic or local complications were observed during or after the examination.

###  Lymphatic Imaging

**FIGURE 4:**
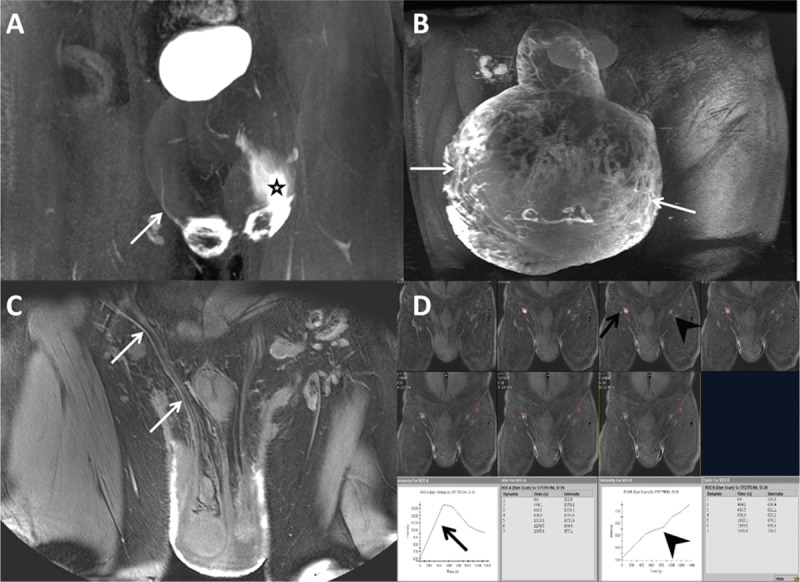
Morphological and functional characteristics of scrotal magnetic resonance lymphography (MRL). (A) Frontal 3D MRL image obtained from an 18-year-old man who had a 2-year history of scrotal swelling accompanied by left limb enlargement owing to primary lymphedema. Only 1 lymphatic vessel could be visualized on the right side of the scrotal wall (arrow), and dermal backflow, an area of progressive dispersion of the contrast media in the soft tissues, showed high signal intensity on left side of scrotal wall (star) and was classified as hypoplasia. (B) MRL image acquired from a 67-year-old man with secondary lymphedema and an 11-month history of a swollen scrotum and penis shows numerous tortuous lymphatic vessels with blurred outlines (arrows), which we classified as hyperplasia. (C) MRL image obtained from a 58-year-old man who had a 2-year history of scrotal swelling owing to secondary lymphedema. The deep lymphatic collector shows a thin linear outline with a high signal intensity along the spermatic cord (arrows). (D) Dynamic MRL obtained from a 37-year-old man with primary lymphedema who had a five-year history of scrotal swelling shows nodal enhancement of the bilateral inguinal lymph nodes in a series images. Peak enhancement could be found in the right inguinal lymph node at 12 minutes after contrast injection (black arrows), whereas no peak enhancement was found in the left inguinal lymph node within the acquisition time (black arrow heads), which we classified as moderate nodal dysfunction.

According to the superficial lymphatic abnormalities observed in the MRL images, 30 (83.3%) patients showed hypoplasia (Figure 4A), and 6 (16.7%) patients showed hyperplasia (Figure 4B). The deep lymphatic vessels, which showed thin high signal lines along the spermatic cord, could be visualized in all 36 patients (Figure 4C). Dermal backflow, an area of progressive dispersion of the contrast media or fluid accumulation in the soft tissues that indicated proximal lymphatic flow obstruction with collateral pathways, correlated well with the occurrence of hypoplasia (Figure 4A).

###  Lymph Node Imaging

The bilateral inguinal nodes could be detected by MRL in all patients. Among the 72 inguinal lymph nodes of the 36 patients investigated, none of the inguinal lymph nodes reached peak enhancement in <9 minutes. There were 41 (56.9%) inguinal lymph nodes that showed peak enhancement (Figure 4D). The mean time to peak enhancement was 19.6 ± 7.8 minutes. There were 31 (43.1%) inguinal lymph nodes that showed no peak enhancement on TIC (Figure 4D). According to the nodal function classification, none of the patients were diagnosed as normal, 7 (19.4%) patients showed mild dysfunction, 26 (72.2%) patients had moderate dysfunction, and 3 (8.3%) patients had severe dysfunction.

###  Treatment Outcomes

Of the 6 patients with hyperplasia, 3 patients each had mild and moderate nodal function. Of the 30 patients with hypoplasia 4 had mild nodal dysfunction, 23 had moderate nodal dysfunction, and 3 patients with severe nodal dysfunction. Therefore, according to our treatment strategies, 7 patients with mild dysfunction underwent CDP. Three patients with hyperplasia and moderate nodal dysfunction underwent lymphovenous microsurgery. Twenty-six patients with hypoplasia and moderate or severe dysfunction were offered surgical excision, but 9 patients with moderate nodal dysfunction rejected surgery in favor of conservative therapy. Thus, there were 14 patients with moderate nodal dysfunction and 3 patients with severe dysfunction who underwent surgical excision (Figure 1).

For the 7 patients with mild dysfunction who underwent CDP, the mean volumes of the scrotum and penis before and after CDP were 6641.5 ± 2619.3 cm^3^ and 2187.2 ± 1829.9 cm^3^, respectively, which was a significant reduction in genitalia volume (*P* < 0.001). For 9 patients with moderate dysfunction who underwent CDP, the mean volumes of the scrotum and penis before and after CDP were 7329.4 ± 1528.4 cm^3^ and 5573.7 ± 1337.6 cm^3^, respectively, which was also a significant volume reduction (*P* < 0.001). The mean volume reduction was 4528.1 ± 2313.3 cm^3^ in patients with mild dysfunction and 1835.4 ± 1097.5 cm^3^ in patients with moderate dysfunction, which was significantly different between the 2 patient groups (*P* < 0.001).

**FIGURE 5:**
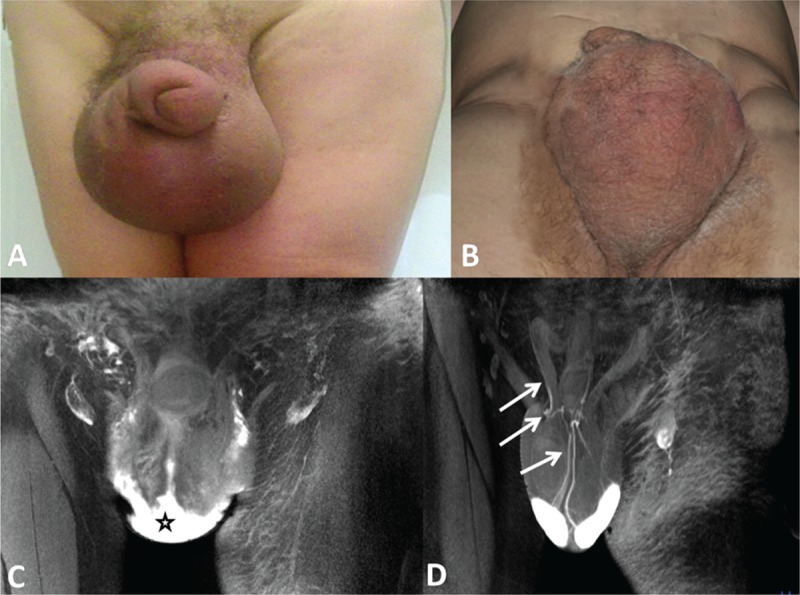
A 66-year-old man with 3-year secondary genital lymphedema underwent the lymphovenous anastomoses technique. (A) Clinical photograph of the scrotum taken before the operation showed evident scrotal and penis lymphedema and skin erythema. (B) Photo of the scrotum taken from the same patient at 6 months after the surgery. Note that the size of scrotum decreased significantly, and the normochromic excess cutaneous tissue disappeared after the edema resolved. (C) Magnetic resonance lymphography (MRL) obtained before microsurgery showed unrecognizable fine lymphatic vessels and dermal reflux (star). (D) Six month later, the patency of the lymphovenous anastomoses was confirmed by MRL and showed the passage of high-intensity signal contrast media from the venule through the anastomosis into the deep lymphatic vessel (arrows).

For the 3 patients who underwent microsurgical operation, the postoperative course was uneventful, and the patients were discharged on postoperative day 5. Six months after the surgery, the scrotum and penis were normochromic with normal consistencies. There were no signs of edema, and the scrotum and penis were significantly reduced in size with normal palpability of the testicles (Figure 5A and B). MRL showed the bilateral patency of the anastomosis in the spermatic cords (Figure 5C and D). During the 3- and 5-year follow-up after surgery, GL did not recur in any patient, and sexual function was not compromised.

**FIGURE 6:**
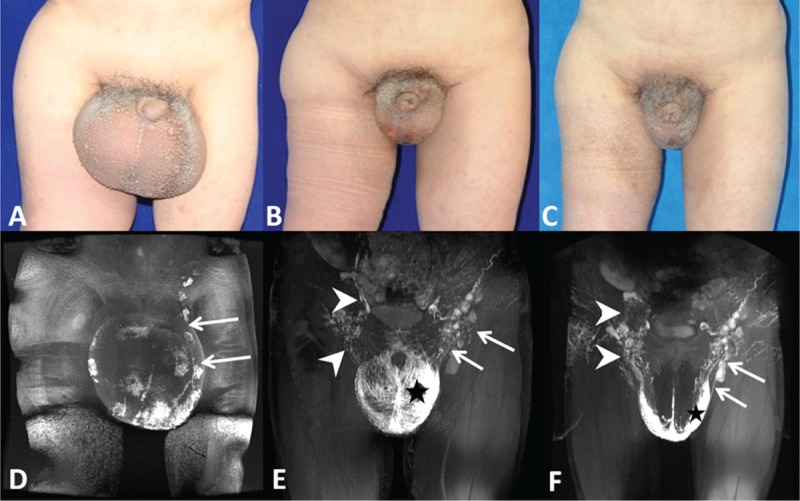
A 28-year-old man with a 13-year primary genital lymphedema underwent surgical excision and scrotal reconstruction. Clinical photographs of the scrotum taken before surgery (A), 3 years (B), and 5 years (C) after surgery showed evident size reduction of the scrotum and penis. (D) Magnetic resonance lymphography (MRL) obtained before surgery showed that the scrotal lymphatic drainage pathway could only be identified on the left side (arrows). The postoperative follow-up MRL at the third (E) and fifth years (F) showed the establishment of new scrotal lymphatic drainage pathways (arrow heads) and reopening of the lymphatic vessels (arrows). The dermal reflex area (star) decreased with postoperative time.

For the 17 patients who underwent partial skin and subcutaneous tissue excision as well as scrotal reconstruction, the surgical excision involved debulking the scrotum in 9 (52.9%) patients and penoscrotal reduction in 3 (17.6%) patients. Hydroceles were encountered and resected in 4 (23.5%) patients. One patient (5.9%) had a postoperative hematoma requiring readmission and revision surgery. All 17 patients had excellent cosmetic results with no recurrence of the scrotal and penile lymphedema at the 3- to 5-year follow-ups (Figure 6A, B, and C). Compared with the preoperative MRL, new formation of scrotal lymphatic drainage pathways or reopening lymphatic vessels could be detected on the follow-up MRL in all patients. The dermal reflex area decreased with postoperative time (Figure 6D, E, and F).

## DISCUSSION

Currently, the determination of therapy modalities for GL is dependent on subjective staging systems that are based on clinical findings. These staging systems have limitations and do not always correlate with underlying physiopathological changes and treatment outcomes.^14^ A major challenge of GL treatment is the high recurrence associated with various treatment modalities. There is no universally accepted treatment strategy and no validated instrument available to assess lymphedema.^15^ By using MRL, new criteria to classify the extent of lymphedema in the external genitalia can be established according to lymphatic vessels and lymphatic drainage in the inguinal node. Based on this classification system, we were able to tailor the therapeutic measures, including CDP, lymphovenous anastomoses, and surgical excision, to different patients, which ensured optimal results for all patients.

The diagnosis and stage of GL is usually made from a clinical history and findings in the physical examination, which is mainly dependent on lymphologist subjective evaluation and their experiences. Three-dimensional MRL is known to provide pathophysiological information regarding lymphostatic diseases of limbs.^6–8^ However, the feasibility and utilization of MRL in patients with GL are rarely reported. Our study showed that MRL could determine the presence and number of lymphatic vessels, the inguinal nodal drainage function, and the obstruction and reflux of lymph of lymphedematous genitalia (Figure 4). MRL provides a morphological assessment and functional classification for treatment strategies. After treatment, MRL provides an accurate assessment of the response to CDP, the patency of microanastomoses and the formation of lymphatic drainage pathways post-reconstruction. Thus, MRL could be an objective and useful tool for helping us to develop a better patient selection process for lymphedema treatment and to better evaluate therapeutic outcomes.

The clinical assessment does not always correlate with hypo- or dysfunction in lymphatic transport. The physical condition of lymphedematous genitalia is related to multiple factors, such as the duration of the disease, etiology, therapy, organ, among others, which do not always correlate with the function of the genital lymphatic system. Some patients were clinically classified as severe stage, whereas the lymphatic drainage function of genital might be mildly affected. We selected patients for conservative treatment based on the lymphatic drainage function of the inguinal lymph nodes. Our results showed that patients with mild dysfunction in lymphatic drainage had a better treatment response than patients with moderate dysfunction after CDP therapy. These results indicated that conservative methods can be effective regardless of the clinical lymphedema stage if associated with the lymphatic drainage function. The less lymphatic drainage function is affected, the better treatment response could be obtained. Our results showed that whether patients had mild or moderate dysfunction in lymphatic drainage, a significant volume reduction of scrotum and penis could be observed after CDP therapy. These results indicated if conservative methods (compression bandaging, manual drainage, therapeutic exercises, and elevation) are performed by experienced therapists, conservative management can lead to a considerable reduction in genital volume.^16^

In present study, we developed a lymphovenous anastomosis technique in patients with hyperplasia and moderate nodal dysfunction based on MRL results. This technique conducted direct anastomoses between the lymphatic collectors of the spermatic funiculus and the vessels tributary to the spermatic veins. By using MRL technique, we were able to rapidly and objectively identify deep functional lymphatic vessels and optimal anatomical locations for lymphaticovenular shunts before making incisions. Mukenge et al^17^ used the indocyanine green approach for lymphography to assess lymphovenous anastomoses for the treatment of secondary GL. Because the infrared camera system those authors used could only detect anatomical structures in the tissue up to 12 mm from the surface,^18^ it would be difficult to find an adequate deep collector for anastomoses, and therefore the surgery duration was increased to 9 hours.^17^ With the help of MRL for the identification of deep functional lymphatic vessels and adjacent small vein, our lymphovenous anastomoses procedure lasted 4 to 5 hours on average (data were not presented). Lymphoscintigraphy is also used to evaluate lymphatic function in the presence of extremity lymphedema.^2^ However, this approach is insufficient to indicate microvascular treatment based on lymphedema because the spatial resolution of lymphoscintigraphy is insufficient to depict tiny lymphatic vessel, which its caliber is less than 1 mm.^19,20^ Our study highlighted that MRL is a very supportive method for guiding bypass surgery and for postoperative follow-up because it can assess lymphatic drainage and ascertain the patency of the anastomosis.

The method of lymphovenous anastomosis directly addresses the pathophysiology of the condition of GL. We chose to perform lymphovenous shunts using the deep lymphatic pathway, which showed that lymphovenous anastomosis is a valuable method for resolving the edematous condition according to MRL findings.^21^ Our findings were consistent with those of Mukenge et al who anastomosed deep lymphatic vessels with the pampiniform venous plexus.^22,23^ Some authors perform anastomoses between the superficial lymphatic vessels and small veins in the subcutaneous tissue.^24,25^ In our opinion, lymphovenous anastomosis using the superficial lymphatic vessels may not improve lymph drainage because of the intima proliferation and function alteration of superficial lymphatic vessels during male GL. To treat male GL with lymphovenous bypasses, based on our present research work, we highly recommend using the deep lymphatic vessel for lymphovenous anastomosis rather than selecting the superficial lymphatic vessels.^23^

As reported in many studies, excisional techniques are most effective for severe forms of GL. We selected patients with moderate and severe nodal dysfunction based on MRL to undergo skin and subcutaneous tissue excision. As we were aware of the different lymphatic functions between the 2 sides of the scrotum before surgery, we preserved scrotal skin from the remaining functional side and used it to reconstruct the scrotum. Because scrotal skin from the functional side was saved, it was not necessary to use split-thickness grafts or thigh pedicle flaps for penile and scrotal reconstruction. In this study, 17 patients who received genital excision showed excellent functional and cosmetic results and marked increases in their life quality. Only 1 patient (5.9%) had a postoperative hematoma, and no recurrences of GL were reported during the 5-year follow-up. The preserved scrotal skin and inguinal lymph nodes had excellent lymphatic drainage functions on the yearly follow-up MRL (Figure 6). Our results are similar to those from other groups who have supported preservation of the posterolateral skin of the scrotum at the perineum because it can be stretched, is rarely involved in the disease, and has a collateral vascular and lymphatic supply.^26^ However, others have found it unwise to preserve the posterolateral skin for scrotal coverage or have found that using split-thickness grafting for scrotal reconstruction is very effective.^2,27^ In this study, we preferred preserved scrotal skin with lymphatic function rather than skin grafts or thigh pedicle flaps to perform primary closure because the former could provide good cosmetic results and full sensation with no risk of scarring and skin graft shrinkage. Moreover, the remnant scrotal skin for scrotum reconstruction does not change the thermal regulation of the testes to interfere testicular function in male patients.^28^

One possible limitation of this study may be the relatively low number of patients enrolled for surgery. However, owing to the prospective nature of this study and considering the potentially uncertain treatment outcomes, few participants underwent surgery. Meanwhile, the strict enrollment criteria for microsurgery further reduced the number of probable patients. In the future, the results should be prospectively collected, ideally in population-based studies.

In summary, MRL can be used to assess the functional severity of lymphedema, identify functioning lymphatic vessels, and enable a lymphologist to preoperatively evaluate and select patients who are best suited for CDP, lymphovenous bypass, or surgical excision. Owing to the minimal invasiveness and lack of radiation, diagnostic follow-up MRL examinations can be performed routinely with little risk to the patient.
